# Asymmetric effects in waveguide systems using PT symmetry and zero index metamaterials

**DOI:** 10.1038/s41598-017-12592-0

**Published:** 2017-09-29

**Authors:** Yangyang Fu, Yadong Xu

**Affiliations:** College of Physics, Optoelectronics and Energy, Soochow University, No.1 Shizi Street, Suzhou, 215006 China

## Abstract

Here we demonstrate directional excitation and asymmetric reflection by using parity-time (PT) symmetric and zero index metamaterials (ZIMs) in a three-port waveguide system. The principle lies on that the field distribution at gain/ loss interface is significantly affected by the incident direction of electromagnetic wave. By taking advantage of the empty volume feature of ZIMs, these asymmetric effects are extended to a more general three-port waveguide system. In addition, by exciting a weak modulated signal in branch port in our proposed design, unidirectional transmission with an unbroken propagation state is achieved, opening up a new way distinguished from the present technologies.

## Introduction

In the past years, asymmetric effects, such as asymmetric transmission, have drawn much attention in numerous research domains. Many schemes to realize these asymmetric effects have been proposed in optics community, including photonic crystals^1–3^, optical nonlinearities^4–7^, magneto-optic effects^8,9^ and metamaterials^10,11^. Furthermore, it has been demonstrated that non-Hermitian Hamiltonians, such as Parity-time (PT) symmetry initially arising from quantum mechanics^12,13^, can possess real spectra and many intriguing physical phenomena have been found, such as power oscillations^14,15^, PT-symmetric laser and absorber^16,17^. In particular, some asymmetric effects in PT symmetric systems have been revealed, such as unidirectional reflectionless^18–21^, asymmetric diffraction^22^ and unidirectional invisibility^23^. Recently it has been suggested that by using matched impedance PT symmetric metamaterials, directional excitation has been observed in a three-port acoustic waveguide system^24^. In the main waveguide, the incident wave undergoes an amplified (decayed) and then decayed (amplified) processes, which is determined by the incident direction. Because the third port (port 3) is opened at the gain/loss layer interface, there will be highly asymmetric field coupled to it. However, such directional excitation can only work for a limited case, i.e., the width of port 3 is much narrower than that in the main waveguide. Otherwise, more scattering will be brought in, and the wider width will give rise to a destroyed wavefront in port 3. In addition, for port 3 with narrower width, only a little wave energy can be squeezed into port 3, which limits the performance of directional excitation.

**Figure 1:**
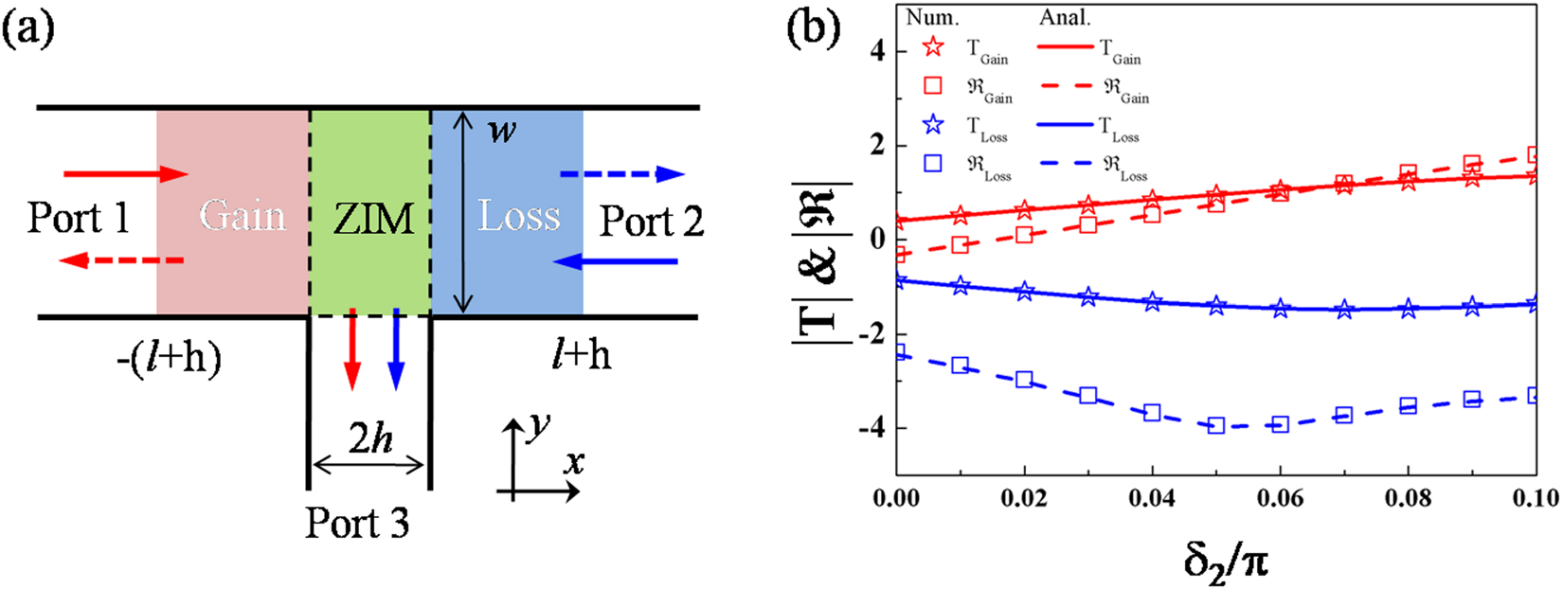
Asymmetric effects in a ZIM waveguide structure with PT symmetric metamaterials. (**a**) The scheme of a ZIM waveguide structure with PT symmetric metamaterials. The ZIM with a length of 2 *h* between the gain and loss layers with lengths of *l* enables to open a rectangular space with arbitrary width for port 3. All the outer boundaries of the three-port waveguide system are perfect electrical conductors (PECs). (**b**) The numerical (symbols) and analytical (curves) relationships between $|{\rm T}|$, $|\Re |$ and *δ*_2_ in a logarithmic scale ($ln(|{\rm T}|)$, $\mathrm{ln}(|\Re |)$), where the red and blue stars (solid curves) are the numerical (analytical) transmission in port 3 for the incident wave from the gain and loss sides, respectively, and the red and blue squares (dashed curves) are the numerical (analytical) reflection in port 1 and port 2 for the incident wave from the gain and loss sides, respectively. *ξ* = 1.2 and ${\delta }_{1}=0.05\pi$ are set for loss and gain media and ${\varepsilon }_{ZIM}={\mu }_{ZIM}=0.01$ are set for the ZIM. For the waveguide configuration, we set $w={w}_{0}$ $l=0.5{w}_{0}$, $h=0.25{w}_{0}$ and $\lambda =0.5{w}_{0}$.

The aim of this work is to reveal how such directional excitation in a three-ports waveguide with PT symmetry is achieved in electromagnetic (EM) wave, which is still absent in optics community. To overcome the drawback of the narrower width of port 3 and obtain a better performance, we employ zero index metamaterials (ZIMs)^21,25–34^ to design a new three-port waveguide structure shown in Fig. 1(a). Due to the qusi-static feature (i.e., the constant field can happen in ZIMs), the introduced ZIMs, providing a relatively large empty volume, can open an arbitrary space at the gain/loss layer interface for port 3. We will demonstrate that the directional excitation and asymmetric reflection can be realized in our proposed ZIM waveguide system with PT symmetry, where the port 3 can possess a wider width. Meanwhile, ZIMs can squeeze the incoming EM wave into the port 3 with the planar wavefront well preserved. More importantly, thanks to the unique property of ZIMs, their external section can be constructed arbitrary, so that such asymmetric effects are extended to a more general three-port waveguide system, which is not accessible in other waveguide structures. Finally, by exciting a weak modulated signal in port 3, we find that unidirectional transmission with an unbroken propagation state of light is achieved in the main waveguide, which stands out from that realized by breaking spatial symmetry^35–38^.

## Results

### Model and Theory

Let us start from the considered three-port waveguide structure shown in Fig. 1(a), where the red and blue sections represent the gain medium and loss medium, respectively, separated by a ZIM layer (green region). The lengths of gain and loss media are *l*; the width of the main waveguide is *w*; the length of ZIM layer is 2 *h*, which can open the same width for port 3. For the gain and loss media, we consider a more general case, with their parameters given as ${\varepsilon }_{G}=\xi \exp (-i{\delta }_{1})$, ${\mu }_{G}=\xi \exp (-i{\delta }_{2})$ and ${\varepsilon }_{L}=\xi \exp (i{\delta }_{1})$, ${\mu }_{L}=\xi \exp (i{\delta }_{2})$, respectively, where *ξ* is the amplitude and ${\delta }_{1,2}\in [0,\,\pi /2]$ is the material phase factor. The ZIM is either mismatched impedance (${\varepsilon }_{ZIM}\cong 0$&${\mu }_{ZIM}\ne 0$ or ${\varepsilon }_{ZIM}\ne 0$& ${\mu }_{ZIM}\cong 0$) or matched impedance (${\varepsilon }_{ZIM}={\mu }_{ZIM}\cong 0$). For discussing conveniently, we consider the matched impedance case for ZIM^28^. All the outer boundaries of the three-port waveguide system are perfect electrical conductors (PEC). A transverse magnetic (TM) wave (its magnetic field along *z* direction) with fundamental mode (transverse electric and magnetic mode) is considered as the source incident from the left (-*x* direction) side or the right (*x* direction) side. The complex index profile of the whole structure guarantees that the Helmholtz equation, i.e., ${\partial }^{2}{H}_{z}/\partial {x}^{2}+\varepsilon (x)\mu (x){{k}_{0}}^{2}{H}_{z}=0$, governing the TM wave propagating along *x* direction, also keep invariant under PT operations, where *k*_0_ = 2*π*/*λ* is wavevector in vacuum and *λ* is the wavelength in air.

To get the propagating characteristic of the EM wave in the three-port ZIM waveguide structure (see Fig. 1(a)), we investigate field distribution in each area. Note that as ${\varepsilon }_{ZIM}\cong 0$, $\mathop{E}\limits^{\rightharpoonup }=i\nabla \times \mathop{H}\limits^{\rightharpoonup }/(\omega {\varepsilon }_{ZIM})$ results in a fact that the magnetic field in ZIM is constant^26,27^, given as *H*_0_. Let us first consider the left incidence, and the corresponding magnetic field in each region can be expressed as,

1a $${H}_{z}^{1}=\exp (i{k}_{0}x)+\Re \exp (\bar{-}i{k}_{0}x),\,x < -(l+h)$$

1b $${H}_{z}^{G}=A\exp (i{k}_{G}x)+B\exp (-i{k}_{G}x),\,-(l+h)\le x\le -h,$$

1c $${H}_{z}^{ZIM}={H}_{0},\,-h\le x\le h$$

1d $${H}_{z}^{L}=C\exp (i{k}_{L}x)+D\exp (-i{k}_{L}x),\,h\le x\le l+h,$$

1e $${H}_{z}^{2}=\Im \exp (i{k}_{0}x),\,x > l+h,$$

1f $${H}_{z}^{3}={\rm T}\exp (-i{k}_{0}y),\,y\le 0$$

where $\Re$ and $\Im$ are the reflection coefficient in port 1 and the transmission coefficient in port 2, respectively, and T is the transmission coefficient in port 3. The corresponding tangential electric field distribution in each region can be calculated by using ${\mathop{E}\limits^{\rightharpoonup }}_{m}=i\nabla \times {\mathop{H}\limits^{\rightharpoonup }}_{m}/(\omega {\varepsilon }_{m})$ (here *m* represents each region). By matching continual boundary conditions and applying the Faraday law $\oint \mathop{E}\limits^{\rightharpoonup }\cdot \,d\mathop{l}\limits^{\rightharpoonup }=-\oiint \mathop{B}\limits^{\rightharpoonup }\cdot \,d\mathop{S}\limits^{\rightharpoonup }$ in the ZIM region^26,27^, all above unknown coefficients can be solved analytically. After some calculations, we get the transmission coefficient in port 3,

2 $${{\rm{T}}}_{Gain}=\frac{{\chi }_{G}}{{{\rm{\Phi }}}_{G}+{{\rm{\Phi }}}_{L}+2h},$$

with

3a $${\chi }_{G}=4{\eta }_{G}w\frac{1}{(1+{\eta }_{G})\exp (-i{k}_{G}l)-(1-{\eta }_{G})\exp (i{k}_{G}l)},$$

3b $${{\rm{\Phi }}}_{G(L)}={\eta }_{G(L)}w\frac{(1+{\eta }_{G(L)})+(1-{\eta }_{G(L)})\exp (2i{k}_{G(L)}l)}{(1+{\eta }_{G(L)})-(1-{\eta }_{G(L)})\exp (2i{k}_{G(L)}l)},$$

where ${k}_{G(L)}={k}_{0}\sqrt{{\varepsilon }_{G(L)}{\mu }_{G(L)}}$ and ${\eta }_{G(L)}=\sqrt{{\mu }_{G(L)}/{\varepsilon }_{G(L)}}$ are the wave vector and the impedance of the gain (loss) medium respectively. Here we use T_*Gain*_ representing the transmission in port 3 for the left incident wave (gain side). For the right incidence, the corresponding T_*Loss*_ can be obtained by performing similar procedure, which is given as,

4 $${{\rm{T}}}_{Loss}=\frac{{\chi }_{L}}{{\Phi }_{G}+{\Phi }_{L}+2h},$$

with

5 $${\chi }_{L}=4{\eta }_{L}w\frac{1}{(1+{\eta }_{L})\exp (i{k}_{L}l)-(1-{\eta }_{L})\exp (-i{k}_{L}l)}$$

Here Φ_*G(L)*_ is the same with that in Eq. 3(b) by replacing *k*_*G*(*L*)_ with −*k*_*G*(*L*)_. In addition, we can also get the corresponding reflection ${\Re }_{Gain}$ and ${\Re }_{Loss}$ for the left incidence and the right incidence, respectively. To explore the underlying asymmetric effects, the corresponding relationship between the transmission ($|{\rm T}|$)/reflection ($|\Re |$) and *δ*_2_ in a logarithmic scale is shown in Fig. 1(b). In calculations, *ξ* = 1.2 and *δ*_1_ = 0.05*π* are set for the loss and gain media, and ${\varepsilon }_{ZIM}={\mu }_{ZIM}=0.01$ are set for ZIM. For the waveguide configuration,*w* = *w*_0_ = 2*λ*, *l* = 0.5*w*_0_ and *h* = 0.25*w*_0_. Both numerical (symbols) and analytical (curves) results in Fig. 1(b) show that the incident wave from the gain side (see the red data) gives rise to a high transmission in port 3 and high reflection in port 1. By contrast, there are extremely low transmission in port 3 and low reflection in port 2 for the incident wave from the loss side (see the blue data). Particularly, when *δ*_2_ = 0.05*π*, there is lowest reflection in port 2, which is caused by the matched impedance in PT symmetric metamaterials (i.e., *η*_*G*_ = *η*_*L*_ = 1). These results show that directional excitation in port 3 and asymmetric reflection in the main waveguide can be realized in our proposed ZIM waveguide with PT symmetry, whatever for a general case (the mismatched impedance of both gain and loss media) or the matched impedance case.

Next we reveal the physical mechanism of these asymmetric effects. For both mismatched impedance and matched impedance cases, they share the similar mechanism. But for the matched impedance case (*δ* = *δ*_1_ = *δ*_2_), it is quite simple and straightforward to discuss the scattering process of EM wave in our proposed waveguide system. In the following, we will focus on this simple case. When *η*_*G*_ = *η*_*L*_ = 1, Equations (2) and (4) are reduced to these simple formulas,

6a $${{\rm{T}}}_{Gain}=\frac{w}{w+h}\exp (i{k}_{G}l),$$

6b $${{\rm{T}}}_{Loss}=\frac{w}{w+h}\exp (-i{k}_{L}l).$$

where ${t}_{G}=\exp (i{k}_{G}l)$ is the transmission at the gain/ZIM interface for the left incident EM wave, ${t}_{L}=\exp (-i{k}_{L}l)$ is the transmission at the loss/ZIM interface for the right incident EM wave, and $\gamma =w/(w+h)$ is the coupling efficiency from ZIM to port 3, which only depends on the geometry sizes. With these formulas, let us examine how directional excitation in port 3 happens for different incident directions. For the left incident EM wave with ${H}_{z}={H}_{in}\exp (i{k}_{0}x)$(H_*in*_ is the amplitude of incident wave), the incoming wave is firstly amplified by the gain medium, and after passing through it, the magnetic field at the gain/ZIM interface is enhanced largely, which is ${H}_{z}^{G}{|}_{x=-h}={t}_{G}{H}_{in}=\tau {H}_{in}\exp (i\phi )$ with $\tau =\exp (\xi {k}_{0}l\,\sin \,\delta )\ge 1$ and $\phi =\xi {k}_{0}l\,\cos \,\delta$. Then the enhanced EM wave ${H}_{z}^{G}{|}_{x=-h}$ enters into ZIM, and ZIM subsequently squeezes the transmitted wave into port 2 and port 3, because of its homogeneous field feature (${H}_{z}^{ZIM}={H}_{0}$). During this process, the coupling efficiency from ZIM to port 3 is not perfect (i.e., *γ* = 1), but *γ* < 1 due to the geometry structures of three ports. Hence, there is some reflection at the gain/ZIM interface, which contributes to $|{\Re }_{Gain}|=(1-\gamma )\tau {H}_{in}$. Finally the transmitted wave in the port 3 is $|{{\rm{T}}}_{Gain}|=\gamma \tau {H}_{in}$. For the right incidence, the incoming wave is firstly attenuated by the loss medium with an attenuated magnetic field at the loss/ZIM interface given as ${|{{\rm{H}}}_{z}^{L}|}_{x=h}={t}_{L}{H}_{in}={\tau }^{-1}{H}_{in}\exp (-i\phi )$. Then the attenuated EM wave enters into ZIM, and similarly ZIM squeezes the transmitted wave into port 1 and port 3, because of the homogeneous field in ZIM. The coupling efficiency from ZIM to port 3 still is *γ* < 1, and the reflection at the loss/ZIM interface is $|{\Re }_{Loss}|=(1-\gamma ){\tau }^{-1}{H}_{in}$. At last the transmitted wave in the port 3 is $|{{\rm{T}}}_{Loss}|=\gamma {\tau }^{-1}{H}_{in}$. Accordingly, the ratio of the EM amplitude in port 3 (the asymmetric reflection) for the left and right incidences is $|{{\rm{T}}}_{G{\rm{ain}}}|/|{{\rm{T}}}_{Loss}|={\tau }^{2}$($|{\Re }_{G{\rm{ain}}}|/|{\Re }_{Loss}|={\tau }^{2}$), which is nothing to do with *γ* < 1. Therefore in our designed system, directional excitation in port 3 and asymmetric reflection in the main waveguide stem from the complex index profile of PT symmetry. To be exact, the performance of these asymmetric effects is determined by this term $\tau =\exp (\xi {k}_{0}l\,\sin \,\delta )$. The merit of ZIM is to open a relatively large space at the gain/loss layer interface for port 3, so that the incoming EM wave can be squeezed into port 3 with the planar wavefront well preserved. Due to the opened ZIM space, the imperfect coupling efficiency *γ* from ZIM to port 3 is determined by *h*, with *γ* → 0 as *h* → ∞. Besides, such imperfect coupling leads to asymmetric reflection, even unidirectional reflectionless^18–21^.

**Figure 2:**
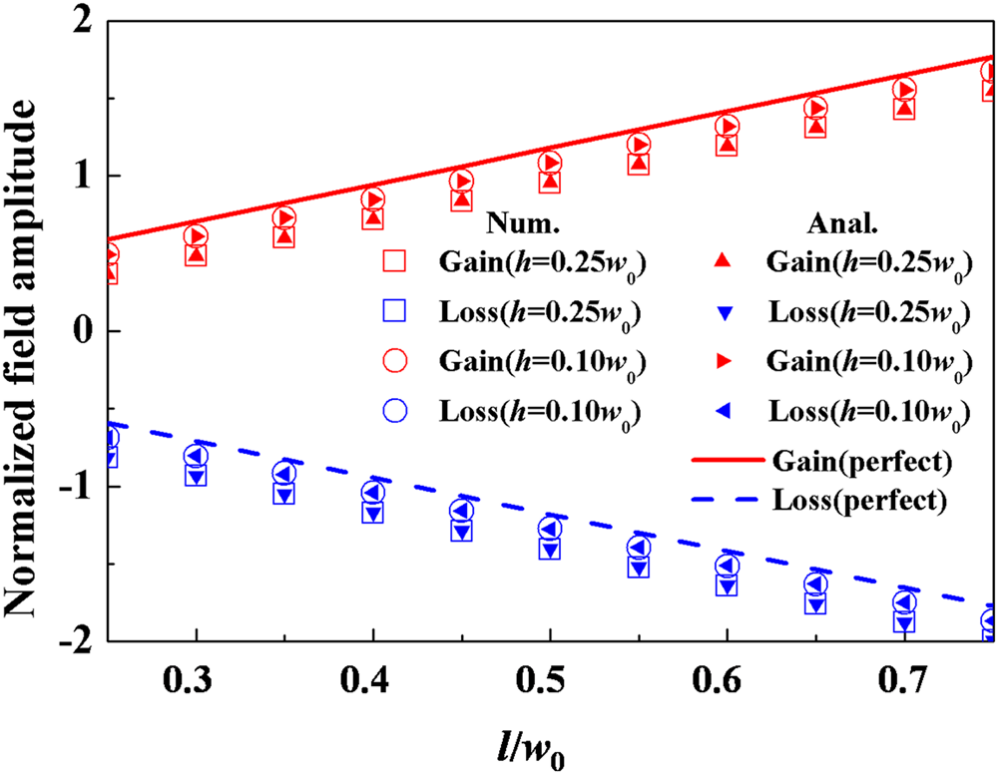
The relationship between the normalized field amplitude in port 3 and the length of PT layers in a logarithmic scale. The red hollow and blue hollow squares (circles) are the numerical results of $h=0.25{w}_{0}$ ($h=0.10{w}_{0}$) for the incident wave from port 1 (gain) and port 2 (loss), respectively. The red solid up-Triangles (right-Triangles) and blue solid down-Triangles (left-Triangles) are the analytical results of $h=0.25{w}_{0}$ ($h=0.10{w}_{0}$) for the incident wave from port 1 (gain) and port 2 (loss), respectively. In addition, the red solid and blue dashed curves are the perfect enhanced and attenuated cases, respectively. In all calculations, we set $\xi =1.2$, $\delta =0.05\pi$, $w={w}_{0}$, $\lambda =0.5{w}_{0}$ and the ZIM is ${\varepsilon }_{ZIM}={\mu }_{ZIM}=0.01$.

Further we investigate the influence of the length *l* and the width *h* on the asymmetric effects. Here we take directional excitation as an example. Figure 2 displays both analytical and numerical relationships between the normalized field amplitude in port 3 (i.e., $\mathrm{ln}(|{\rm{T}}|/{H}_{in})$) and the length of PT layers for two different widths of port 3. The analytical results are calculated by $\mathrm{ln}(|{{\rm{T}}}_{Gain}|/{H}_{in})=\,\mathrm{ln}(\gamma \tau )$ for the incident wave from the gain side and $\mathrm{ln}(|{{\rm{T}}}_{L{\rm{oss}}}|/{H}_{in})=\,\mathrm{ln}(\gamma {\tau }^{-1})$ for the incident wave from the loss side. For *h* = 0.25*w*_0_, the analytical transmission for the left incidence (gain side) is shown by the red solid up-triangles, while it is shown by the blue solid down-triangles for the right incidence (loss side). The increasing length of PT layers leads to the enhanced (attenuated) fields for the left (right) incidence, with a dramatic contrast between them. The reason is that the longer length *l* gives rise to longer enhanced (attenuated) process in the gain (loss) layer. Moreover, the similar tendency can be seen for port 3 with a narrower width of *h* = 0.10*w*_0_. The only difference is that for the same length *l*, the enhanced/attenuated fields in the case of *h* = 0.10*w*_0_ are larger than these in the case of *h* = 0.25*w*_0_. It is because for port 3 with a narrower width, the coupling efficiency $\gamma =w/(w+h)$ is larger. As a result, these enhanced or attenuated field amplitudes for port 3 with a narrower width will more approach the perfect cases (see the red solid curve for the enhanced field; the blue dashed curve for the attenuated one in Fig. 2) that are calculated based on *γ* = 1. It is noted that for both cases, the gap between the enhanced and attenuated fields is unchanged for a fixed length of PT layers, because the ratio $|{{\rm{T}}}_{Gain}|/|{{\rm{T}}}_{Loss}|={\tau }^{2}$ is constant and only dependent on PT symmetric parameters. In addition, the corresponding numerical calculations by using COMSOL Multi-physics software based on finite elements methods are also displayed in Fig. 2, where the numerical results agree well with the analytical results. The similar results for asymmetric reflection can be also obtained by observing the analytical equations: $|{\Re }_{Gain}|/{H}_{in}=(1-\gamma )\tau$ and $|{\Re }_{Loss}|/{H}_{in}=(1-\gamma ){\tau }^{-1}$. Therefore, directional excitation in port 3 and asymmetric reflection in the main waveguide are well revealed in a three-port ZIM waveguide with PT symmetry.

### Numerical verification for directional excitation and asymmetric reflection

**Figure 3:**
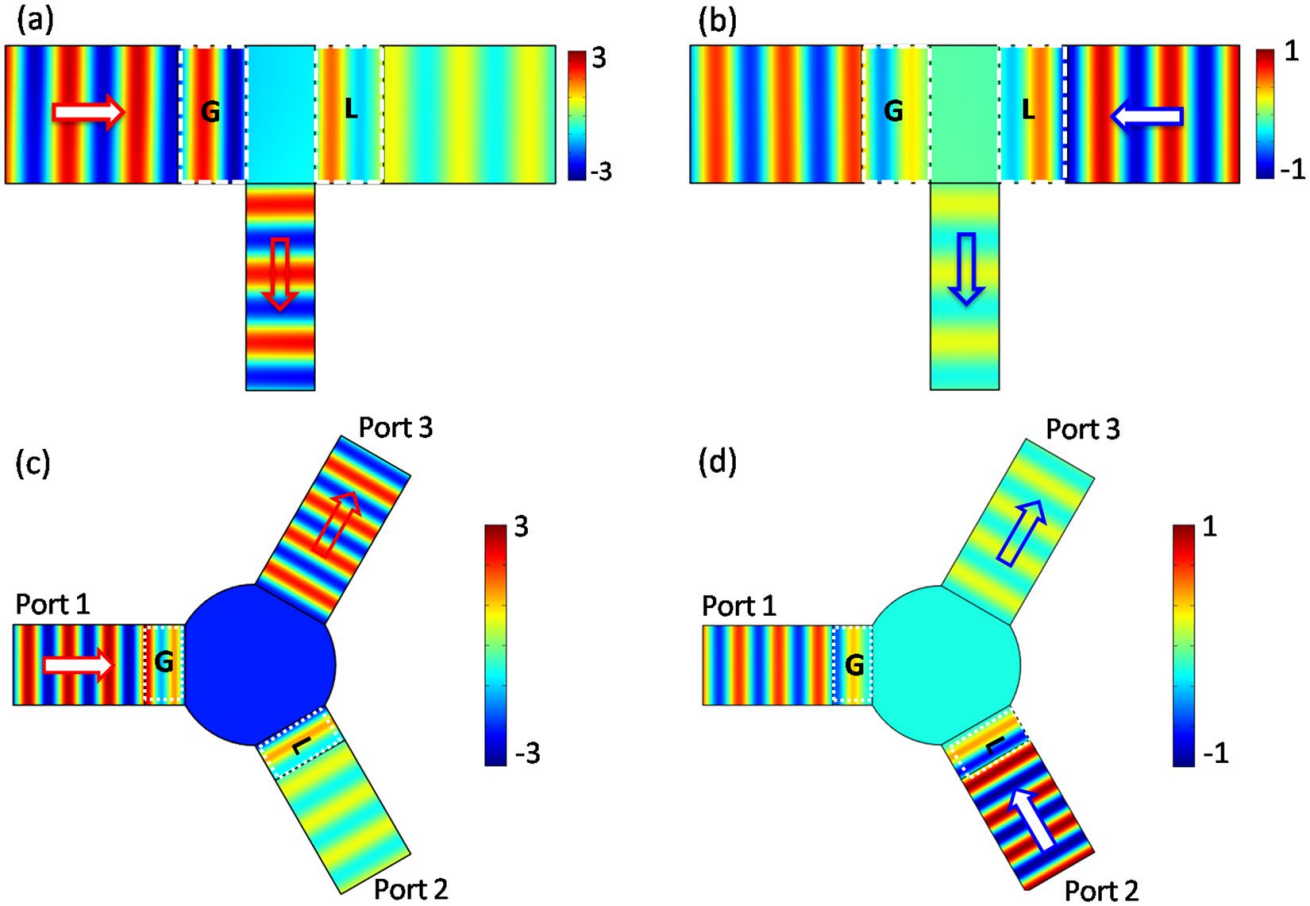
Directional excitation and asymmetric reflection from the proposed three-port ZIM waveguide with PT symmetry. (**a**) and (**b**) are the corresponding magnetic field patterns in a three-port waveguide system for the incident wave from port 1 and port 2, respectively. In the simulations, we set *ξ* = 1.2, *δ* = 0.05 *π*, *w* = *w*_o_, *λ* = 0.5*w*_o_ *l* = 0.5*w*_0_, *h* = 0.25*w*_0_ and the ZIM is ${\varepsilon }_{ZIM}={\mu }_{ZIM}=0.01$. (**c**) and (**d**) are the corresponding field patterns in a three-port ZIM waveguide system for the cases of the incident waves from port 1 with gain medium and port 2 with loss medium, respectively, where the area of ZIM is constructed into circle cross section (*r* = *w*_0_). The widths of these ports are *w*_0_. In the simulations, other parameters are identical with these in (**a**) and (**b**).

To illustrate the above asymmetric effects, we carry out the numerical simulations to obtain the field patterns by using COMSOL Multiphysics. In the simulations, we set *l* = 0.5*w*_o_ and *h* = 0.25*w*_o_, and the other parameters are identical with these in Fig. 2. Figures 3(a) and (b) display the simulated magnetic field patterns, which correspond to the incident wave from port 1 (gain) and port 2 (loss), respectively. By observing the field distribution in Fig. 3(a), for the incident wave with unity amplitude from port 1, the enhanced field amplitude larger than unity can occur in port 3 with an unbroken planar wavefront. On the contrary, it is attenuated field amplitude in port 3 for the incident wave from port 2 (see Fig. 3(b)). In addition, as port 3 has a wider width, there is imperfect coupling from ZIM to port 3, which leads to unidirectional reflectionless^18–20^. For the incident wave from the gain side, due to the imperfect coupling, some of the enhanced wave will be reflected back through gain medium again ($|{\Re }_{Gain}|\gg 0$), which results into stronger reflection in port 1 (see Fig. 3(a)). While for the incident wave from the loss side, some of the attenuated wave will be reflected back through loss medium again ($|{\Re }_{L{\rm{o}}ss}|\to 0$), giving rise to almost zero reflection in port 2 (see Fig. 3(b)). Alternatively, thanks to the unique EM properties of ZIMs, i.e., the constant field can take place in ZIMs, they can be constructed into an arbitrary shape with optional dimension, showing much flexibility in potential applications. For instance, we can design a three-port waveguide structure connected by the ZIM with circle cross section (see Fig. 3(c) and (d)). The port 1 and port 2 are equipped with gain and loss parts of PT symmetric metamaterials, respectively; the port 3 is an empty one. The widths of three ports are the same (*w* = *w*_0_) and they are connected by a ZIM with a radius of *r* = *w*_0_. By comparing the simulated magnetic field patterns in Fig. 3(c) and (d), the field amplitudes in port 3 are greatly affected by the incident waves from different ports, and both directional excitation and unidirectional reflectionless are excellent.

### Unidirectional transmission

**Figure 4:**
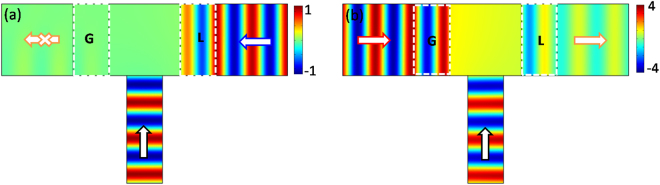
Unidirectional transmission by introducing a modulated wave in port 3. (**a**) and (**b**) are the corresponding simulated magnetic field patterns for the cases of the incident waves from port 2 with loss and port 1 with gain, respectively. In plots, to ensure better destructive interference in ZIM, the cross section of ZIM is designed into square one with a side length of *w*_0_.

Another potential application of our proposed design is that unidirectional transmission with an unbroken propagation state in the main waveguide can be achieved and controlled by using light or wave. Here we take the geometry configuration in Fig. 1(a) for discussion. In this scenario, port 3 is no longer a passive port, but a controlled port to offer reference signal. As shown in Fig. 3, the magnetic field in ZIM is highly asymmetric for different incident directions. In particular, for the incident wave from the loss side, the field at loss/ZIM interface is ${H}_{z}^{L}{|}_{x=h}={\tau }^{-1}{H}_{in}\exp (-i\phi )$, which is quite weak. To ensure a better destructive interference in the ZIM, a weak reference EM wave with ${{H}_{z}}^{ref.}=-({w}_{0}/2h){H}_{z}^{L}{|}_{x=h}$ can be applied in port 3. In this way, when the incident wave from port 2 has an attenuated magnetic field at the loss/ZIM interface equal to ${H}_{z}^{L}{|}_{x=h}$. Meanwhile, the reference wave from port 3 reaches the ZIM surface with a magnetic field ${{H}_{z}}^{ref.}$, spreading along the ZIM surface with an ultimately stable magnetic field $-{H}_{z}^{L}{|}_{x=h}$. Finally, the incident waves from port 2 and port 3 undergo destructive interference, leading to almost zero transmission in port 1. Numerical simulation is carried out to verify this result shown in Fig. 4(a), where there is almost zero transmission in port 1 for the incident wave from port 2 together with the modulated wave from port 3. While for the incident wave coming from port 1, an enhanced magnetic field with ${H}_{z}^{G}{|}_{x=-h}=\tau {H}_{in}\exp (i\phi )$ has interaction with the modulated wave from port 3. As the modulated wave is much weaker than ${H}_{z}^{G}{|}_{x=-h}$, the constant field in ZIM is almost equal to ${H}_{z}^{G}{|}_{x=-h}$ after the interference of these two waves. Eventually, after the constant field passes through the loss medium, there is a higher transmission in port 2. Figure 4(b) shows numerical simulation, where the transmission coefficient is about 73%. Moreover, by decreasing the width of port 3, the coupling coefficient *γ* will increase, and the transmission in port 2 can be further increased. Therefore, by introducing a weak modulated wave in port 3, unidirectional transmission is realized with the transmitted planar wavefront well preserved. This effect is not accessible in previous literatures^35–38^ where these unidirectional behaviors are obtained at the price of destroying wavefronts of the transmitted waves by breaking the spatial symmetry.

## Discussion

By combining PT symmetric metamaterials and ZIMs, directional excitation and asymmetric reflection have been well realized in our proposed waveguide structure (even in a more general case), which has more advantages than that in the acoustic design. What’s more, by modulating a special weak signal in the branch waveguide, unidirectional transmission with an unbroken propagation state is achieved in our design, which is innovative in comparison to these realized by breaking spatial symmetry^35–38^. The underlying mechanism of the above asymmetric effects is well explained based on the matched impedance case of PT symmetry, which shares the similar mechanism with that in the mismatched case. These asymmetric effects can be further explored in other non-Hermitian systems, such as passive systems with loss, the general PT symmetric systems^39^, non-ideal PT symmetric systems^40^ (loss and gain are imbalanced) and conjugate metamaterials^41^. Although the wave scattering problem in these systems is more complicated, elaborate optimization for highly asymmetric effects can be completed by adjusting the parameters of loss/gain, the configuration of structure, the size, and so on. For experiments, the required ZIM can be realized by layered structures^28^, photonic crystals^29^. Although PT symmetric metamaterials are quite difficult to realize, the experimental process in non-Hermitian optics^42^ offers some promise in future. In addition, the active acoustic PT symmetric metamaterials^43,44^ have drawn much attention, and therefore it is possible to realize our design in acoustic domain by using active acoustic modulation.
